# Prevention of Sudden Death in Sports: A Global and Multidisciplinary Observatory for Scientific Research and Knowledge Transfer (PREMUBID)

**DOI:** 10.31083/j.rcm2401012

**Published:** 2023-01-06

**Authors:** Nuria Garatachea, Esther Pueyo, Thijs M.H. Eijsvogels

**Affiliations:** ^1^Growth, Exercise, NUtrition and Development (GENUD) Research Group, University of Zaragoza, 50009 Zaragoza, Spain; ^2^Department of Physiatry and Nursing, Faculty of Health and Sport Science (FCSD), University of Zaragoza, 22002 Huesca, Spain; ^3^Instituto Agroalimentario de Aragón (IA2), Centro de Investigación y Tecnología Agroalimentaria de Aragón (CITA), Universidad de Zaragoza, 50013 Zaragoza, Spain; ^4^Biomedical Signal Interpretation and Computational Simulation (BSICoS), Aragón Institute for Engineering Research (I3A), IIS Aragón, University of Zaragoza, 50018 Zaragoza, Spain; ^5^CIBER de Bioingeniería, Biomateriales y Nanomedicina, CIBER-BBN, 28029 Madrid, Spain; ^6^Radboud Institute for Health Sciences, Department of Physiology, Radboud University Medical Center, 6500 HB Nijmegen, The Netherlands

**Keywords:** sudden cardiac death, sport, research, network

## Abstract

**Background:**

The health benefits of sports and exercise training are well known. However, an acute bout of exercise transiently increases the risk of sudden cardiac death (SCD). To minimize the cardiovascular risks of exercise, more insight into the prevention and causes of SCD is needed.

**Methods:**

The observatory for the prevention of sudden death in sports, PREMUBID, was created with the aim of fostering research to assess the benefits and risks of exercise at different volumes and intensities and to get insight into the underlying mechanisms of potentially cardiac (mal) adaptations.

**Results:**

The observatory gathers researchers from a wide range of disciplines working at institutions in Europe and North America. The guiding principles of PREMUBID are to broaden the understanding of SCD in sports, strengthening collaborative research across the globe, and to develop, implement and evaluate robust pre-participation screening and emergency care strategies to further reduce the number of fatal cardiac events in sport events. During the inaugural meeting of the observatory, members and affiliated researchers discussed possibilities to initiate collaborative research projects and to exchange staff and students to share information and practices to prevent SCD. The final goal is to translate the obtained knowledge to understandable messages for the general population and healthcare workers to ensure that the population at large benefits from it.

**Conclusions:**

The PREMUBID consortium aims to produce novel knowledge and insights in SCD prevention, in order to maximize the health benefits associated with acute and long-term exercise training.

## 1. Introduction

The health benefits of sports and exercise training are widely acknowledged. Compelling evidence supports a strong and curvilinear association between regular physical exercise and a risk reduction for all-cause mortality, cardiovascular mortality and sudden cardiac death (SCD) [1, 2, 3]. Nevertheless, sports participation is known to transiently increase the risk for sudden cardiac arrest and SCD, which has an extensive social impact due to its apparent preventable nature [4]. SCD incidence varies widely across studies, with an event rate ranging from 0.31 to 2.1 per 100,000 person-years [5, 6, 7, 8] depending on age, sex [9] and sport level participation [5] of the population. The majority of exercise-related SCDs occur during recreational sports rather than in organized competitive sports and most of these SCDs occur in adults above 35 years of age [10]. The relative risk of SCD during and up to one hour after vigorous exertion is higher when compared with rest or more moderate activities. For example, a 3-fold increase in risk was reported in a retrospective case-crossover study involving both men and women (n = 206) [11], whereas a 17-fold increased risk was found in another cohort including only men (n = 21,481) [12]. Individuals with the lowest levels of regular physical activity (i.e., <once per week) had the highest SCD risk during and shortly after vigorous exertion [12, 13]. Limited data are available from studies involving only women to analyze the risk of exercise-related SCD during or after vigorous versus moderate exercise and as a function of their habitual physical activity level [2].

Atherosclerotic coronary artery disease is the most common cause of SCD in middle aged and older athletes [5, 11, 14], whereas hereditary or congenital heart diseases have most commonly been associated with exercise-related SCD in young athletes (<35 years old) [14]. Hypertrophic cardiomyopathy (HCM), arrhythmogenic right ventricular cardiomyopathy (ARVC), long QT syndrome (LQTS) and catecholaminergic polymorphic ventricular tachycardia are examples of such underlying conditions. Although HCM has been traditionally considered the most common cause of exercise-related SCD in young individuals, marked discrepancies exist between studies, which could be explained by multifactorial reasons related to geographical differences, HCM diagnostic criteria, study methodologies, proportion of performed post-mortem examinations and experience of forensic pathologists [15]. Recent studies suggest an apparent change in the prevalence of exercise-related SCD causes from HCM as the predominant cause towards others causes of death associated with a structurally normal heart [16, 17]. Knowledge of the causes behind exercise-related SCD in young and adult individuals may have important implications for the strategies implemented to prevent these events. In terms of pre-participation screening, methods based on electrocardiography (ECG) could be useful to detect some hereditary and congenital heart diseases, while (advanced) image modalities could be recommended to identify the presence and severity of occult coronary artery disease [17]. Although national medical societies advocate for cardiac screening of athletes prior to participating in sports [18], there is an ongoing debate regarding the optimal and most cost-effective strategy [19, 20]. Additionally, it is well-accepted that time to cardiopulmonary resuscitation (CPR)/defibrillator shock is key for survival [21, 22], which should encourage all individuals that work at sport events (e.g., medical personnel, trainers, coaches, referees) to be properly trained in recognizing the symptoms of sudden cardiac arrest, in performing CPR and in the use of automatic external defibrillators [23].

## 2. PREMUBID: An Observatory for the Prevention of Sudden Death in Sports

**Fig. 1.**
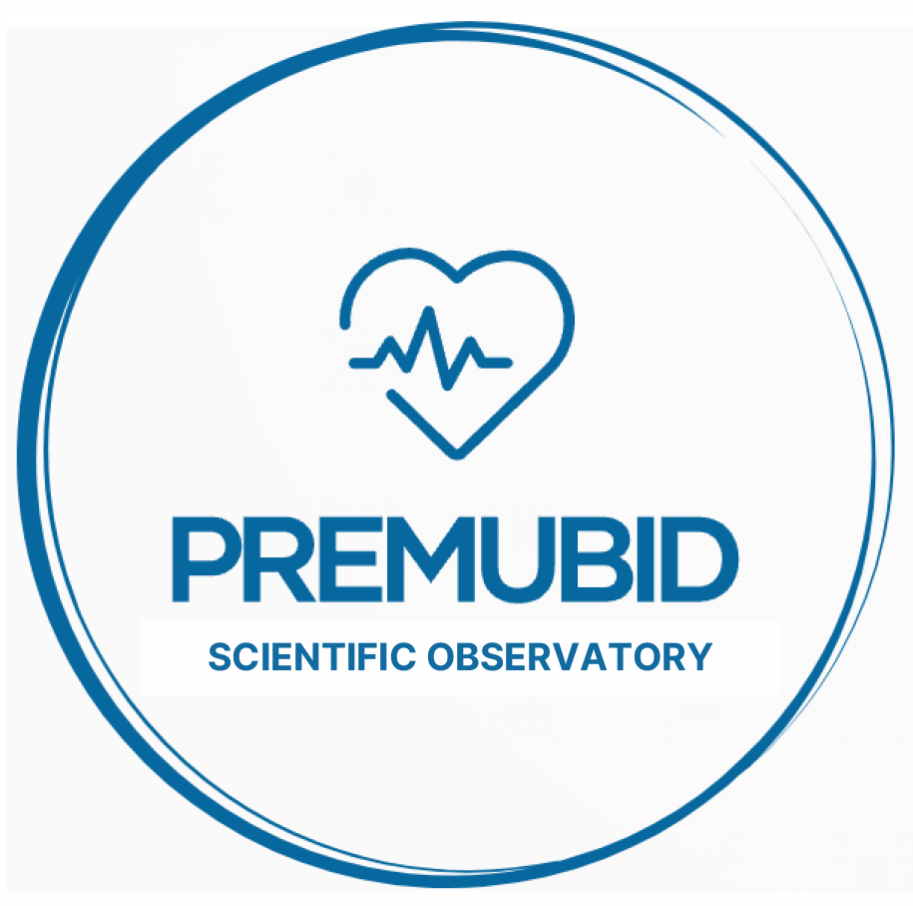
**PREMUBID logo**.

Based on the importance of research to understand the benefits and risks of exercise at different volumes and intensities and to get insight into the underlying mechanisms, a group of sport researchers developed a roadmap for the creation of a research network under a call from the Spanish High Council for Sport. Their goal was to build an international framework able to harness the strong desire for collaboration within the sports cardiology community and to identify barriers that impede multicenter partnerships. In 2021, the observatory ‘Prevention of sudden death in sports: a global and multidisciplinary observatory for scientific research and knowledge transfer’ (PREMUBID, a Spanish acronym for *Observatorio Científico en Red para el Estudio y la PREvención de la MUerte SúBIta en el Deporte*) was founded (PREMUBID, Fig. 1). PREMUBID aims to serve as a model organization to link geographically distant research groups to foster research on exercise-related SCD.

**Fig. 2.**
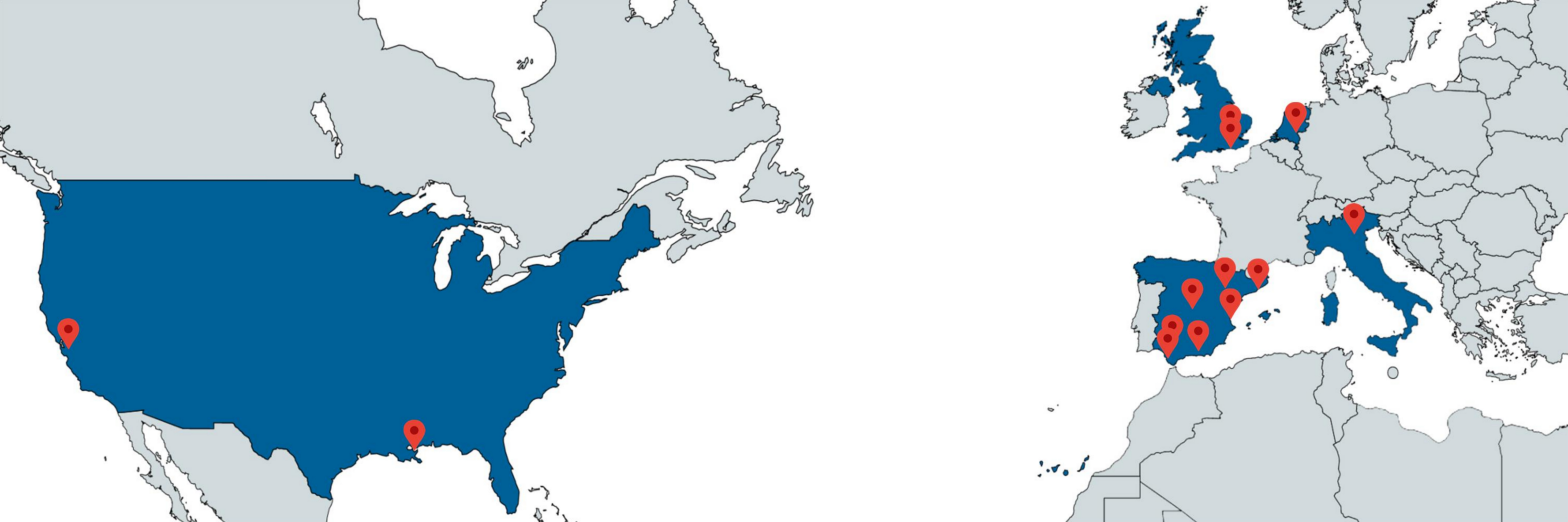
** PREMUBID member sites**.

At the inauguration, PREMUBID convened a 42-member multidisciplinary consortium consisting of sport scientists, cardiologists, sport medicine specialists, exercise physiologists, sport coaches, managers and bioengineers, with members from Spain, the Netherlands, Italy, Great Britain and the United States of America (Fig. 2). Researchers, clinicians and representatives of sport- and health-related organizations are welcome to join the consortium and can contact the authors to apply for membership through the PREMUBID website (https://www.premubid.info).

**Fig. 3.**
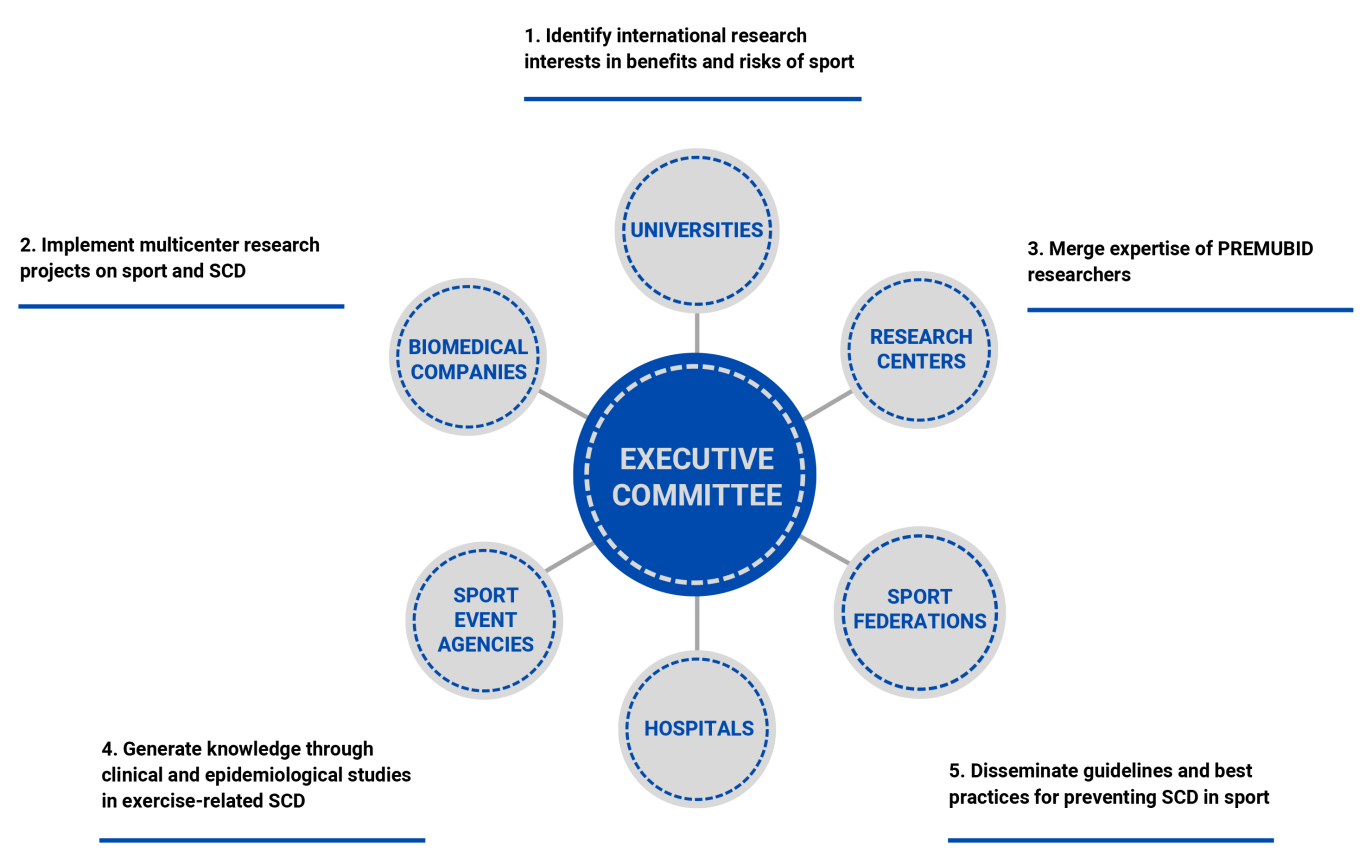
**Organizational chart of PREMUBID including its 5 key goals**.

Each PREMUBID member is expected to attend the biannual scientific meeting and communicate about new study proposals to gauge interest and extend the possibility to other members to participate in new initiatives. PREMUBID Executive Committee includes four members: chair, vice-chair, secretary and treasurer. A research coordinator from each geographical site is appointed, and serves as the primary contact for the Executive Committee. The Executive Committee (Fig. 3) is responsible for planning and setting the agenda for the PREMUBID scientific meeting, communicating regularly with all members about new studies, funding opportunities, conducting reviews, monitoring ongoing studies and developing and disseminating guidelines for preventing SCD in sports. A core principle of PREMUBID is its open membership without restrictions by discipline, specialty or research training. All members can put forth new ideas to develop full proposals for funding or share databases. The following network goals are established as guiding principles: (1) Identify shared international research interests in SCD in sport. (2) Establish and test efficient and flexible ways of developing and implementing multicenter research projects. (3) Pool the expertise of researchers and develop cohesiveness between centers involved in PREMUBID. (4) Generate knowledge through clinical and epidemiological studies in sport-related SCD. (5) Develop and disseminate guidelines and best practices for preventing SCD in sports.

## 3. Inaugural PREMUBID Meeting

The single-day inaugural meeting was held at the University of Zaragoza (Huesca, Spain). The onsite and online program [24] offered sessions for researchers, clinicians, students and non-governmental organizations about vigorous exercise and SCD. In the inaugural conference, Dr. Eijsvogels [25, 26, 27] reviewed recent studies covering the acute and long-term cardiovascular consequences of extreme exercise on heart health. Next, Dr. Bailón and Dr. Pueyo [28, 29, 30, 31, 32, 33] described biomedical engineering methods for the analysis of ECG recordings from athletes and the characterization of risk markers of SCD. The conference closing was presented by Dr. Sanchis-Gomar [34, 35, 36, 37, 38], who discussed how exercise contributes to cardiovascular health, including novel findings on the impact of exercise intensity and duration on cardiac function.

During the meeting, PREMUBIBD members and other researchers shared information and exchanged knowledge about best practices to prevent and manage SCD. Attendees showed common interests in high-quality research and dissemination to achieve safe sport participation in varied environments and during competitive events throughout the world. They discussed possibilities to initiate a research project with ambitious objectives while achievable on a low-budget, voluntary basis and to exchange staff and students to stimulate learning, exchange skills and knowledge and undertake joint studies.

Strong working relationships are beginning to emerge among PREMUBID members. These relationships are expected to provide opportunities to answer relevant questions on SCD in sports through large collaborative research studies that can only be conducted in an international context involving large numbers of individuals, both male and female, young and old and amateur and elite athletes. Translating the obtained knowledge into sports practice is deemed fundamental to ensure that the whole population benefits from the high-quality research conducted by PREMUBID scientific observatory.

## 4. Challenges and Vision for the Future

A core element for PREMUBID success, and a constant challenge in research, is funding. Large grants are required to conduct multicenter studies. Although the observatory has no annual funding budget yet, it is committed to promote the submission of high-quality studies to research calls. Also, PREMUBID aims to work with all involved investigators to broaden the understanding of vigorous exercise’s acute and chronic cardiovascular complications, which can lead to novel research and opportunities for funding.

Currently, PREMUBID comprises representatives from Europe and North America. PREMUBID is committed to encourage and support involvement from other geographical regions. Such global collaboration is expected to result in more large-scale and generalizable knowledge of SCD in sports.

The observatory will work closely with healthcare professionals and knowledge mobilization groups to encourage dissemination and transfer of knowledge to achieve the ultimate aim of improving the care of athletes. Updates in PREMUBID research priorities will be defined after consultation with a broad range of stakeholders, including researchers, public health practitioners and sport event managers. The distinct roles of the involved members will be clearly identified to integrate research into a translational pathway.

## 5. Conclusions

Our understanding of the risks of (vigorous) exercise on SCD is advancing, but further research is required to improve the knowledge of the causes underlying exercise-related SCD. Such insight is expected to serve as a basis for the development of methods to prevent the occurrence of SCD in sport. The recently founded PREMUBID observatory has 5 key priorities with the aim to broaden the understanding of SCD in sports, strengthening a more collaborative research across the globe and propose more robust pre-participation screening and emergency care strategies to reduce the number of fatal cardiac events in sport activities.
